# Spatial variation in diatom abundance and composition in Biwase Bay and Hamanaka Bay (Eastern Hokkaido, Japan), with reference to environmental features

**DOI:** 10.7717/peerj.13705

**Published:** 2022-07-27

**Authors:** Hyojin Ahn, Miho Ito, Naoko Kouchi, Kentaro Watanabe, Hiroya Abe, Tomonori Isada, Masahiro Nakaoka

**Affiliations:** 1 Akkeshi Marine Station, Hokkaido University, Akkeshi, Hokkaido, Japan; 2 Faculty of Fisheries Science, Hokkaido University, Hakodate, Hokkaido, Japan; 3 Department of Biological Sciences, Hokkaido University, Sapporo, Hokkaido, Japan; 4 Kiritappu Wetland National Trust, Hamanaka, Hokkaido, Japan; 5 Graduate School of Environmental Science, Hokkaido University, Sapporo, Hokkaido, Japan

**Keywords:** CDOM, Chl. a concentration, Diatom composition, Coastal Oyashio Water, River discharge, Terrestrial input

## Abstract

This study aims to examine the spatial variation of diatom abundance and composition along the nearshore areas of Biwase Bay and Hamanaka Bay, eastern Hokkaido. Terrestrial input *via* Kiritappu Wetland is expected to affect variation and composition differently depending on the position of the two bays. We conducted an oceanographic survey in June 2014 to measure seawater temperature, salinity, colored dissolved organic matter (CDOM) absorption, nutrient concentrations, and total and size-fractionated chlorophyll (Chl) *a* concentration at 11 stations of the shallowest (<5 m) parts of the bays. These were grouped into four areas (Areas 1 and 2 in Biwase Bay, and Areas 3 and 4 in Hamanaka Bay) based on the distance of the location from the wetland outlet (nearest in Area 1 to the farthest in Area 4). Diatoms are the major primary producers in the water column. Therefore, we also determined genus level cell abundance and diversity of diatoms to compare similarity among areas. Sea surface temperature was the lowest at Area 4, whereas sea surface salinity was the lowest at Area 1. The contribution of CDOM absorption, an indicator of wetland-influenced river discharge, and silica concentration was highest at Area 1. Total amount of nitrite and nitrate concentrations was the highest at Area 4. Total amount of Chl *a* concentration was also lowest in Area 1. Our size-fractionated Chl *a* results revealed that while the size composition of phytoplankton varied among areas, micro-sized (>10 µm) phytoplankton were predominant in Area 4. Finally, diatom composition at the genus level differed greatly among areas. Pennate diatoms were predominant in Areas 1 and 2, but centric diatoms dominated in Areas 3 and 4. Our results suggested great spatial variability in oceanographic conditions among areas, with less influence of wetland and more influence of Coastal Oyashio Water based on distance from the wetland outlet. Diatom composition showed geographical division between Biwase and Hamanaka Bays.

## Introduction

Primary productivity in coastal ecosystem supports various components of marine biodiversity and ecosystem functions (terHorst & Munguia, 2008). This affects not only higher trophic levels in the pelagic layers (Sigman & Hain, 2012), but also benthic consumers including important fisheries targets (*e.g*. bivalves, decapod crustaceans) (Caswell & Coe, 2013; Doherty-Bone et al., 2019). Coastal ecosystems are affected by oceanic dynamics, terrestrial input and geographical (topographical) structures, which make spatial variation in communities and production greater and more complex than offshore pelagic ecosystems (Burke et al., 2000).

Eastern Hokkaido is predominantly influenced by Coastal Oyashio Water (COW) which brings low temperature and low salinity (Isoda & Kishi, 2003; Kono et al., 2004). Spring phytoplankton blooms occur every year in the COW, supporting high productivity and ultimately various seafood resources (Kasai et al., 1997). Although the extent of global coastal wetlands (Hu, Niu & Chen, 2017) is seeing overall declines due to various anthropogenic activities, pristine freshwater/brackish wetlands remain in eastern Hokkaido (Scott et al., 1995). These buffer against the direct river discharge of terrestrial matter through rivers (Burke & Gibbons, 1995). Continued protection and conservation of wetlands may benefit coastal productivity and sustainability. Therefore, surveys of areas that have coastal wetlands are important.

**Figure 1:**
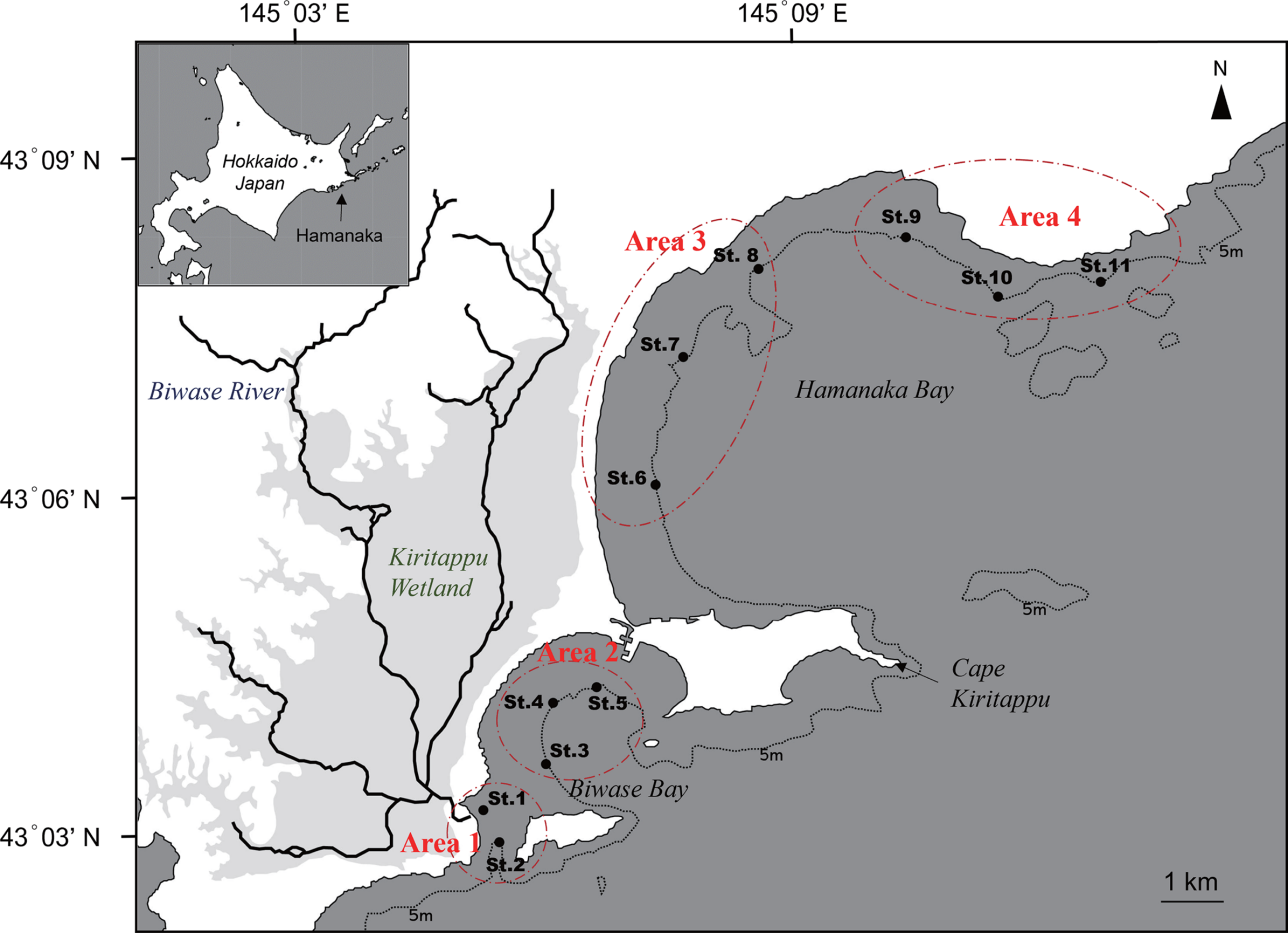
Map of Biwase Bay and Hamanaka Bay surrounding Kiritappu Wetland, Hamanaka Town, eastern Hokkaido, Japan. Closed black circles indicate sampling stations. Stations were divided into four areas (Areas 1–4).

Hamanaka town is a key fisheries town in eastern Hokkaido (Shikida et al., 2009). The coastal area of Hamanaka town is divided into Biwase Bay and Hamanaka Bay, both of which face Kiritappu Wetland (Fig. 1). Kiritappu Wetland is the third largest wetland of Japan (3,168 ha) and internationally protected by the Ramsar Convention (Ito, 1999). Biwase Bay is a semi-enclosed bay directly impacted by river-discharge of Biwase River running through Kiritappu Wetland. Hamanaka Bay is open to the Pacific and is therefore more affected by COW than the wetland. Spatial comparisons of these two bays will give insight into how much the effects of wetland extends to coastal areas. Ba et al. (2018) investigated the environmental conditions and origins of organic matter of this area to infer the role of the wetland to the coastal environment. However, impacts on pelagic productivity and its component has not been precisely tested.

The main objective of this article is to examine spatial variation of diatoms along nearshore areas of Biwase and Hamanaka Bays that are implied to display terrestrial effects from Kiritappu Wetland. We conducted *in situ* surveys measuring seawater temperature, salinity, nutrients concentration, and colored dissolved organic matter (CDOM) absorption as indicators of wetland-influenced river discharge at the surface to investigate the spatial variation in abiotic coastal water properties in nearshore areas. In addition to observations on diatom taxa composition, we measured both total and size-fractionated chlorophyll (Chl) *a* concentrations to act as biotic environmental factors of the study area.

## Materials and Methods

### Water sampling

Seawater sampling was conducted at a total of 11 stations in Biwase and Hamanaka Bays on June 23, 2014 in the timing of flood tide (Fig. 1). Sampling stations were divided into four areas as follows: Area 1, Biwase Bay near the Biwase River mouth (St.1-2); Area 2, Biwase Bay facing Kiritappu Wetland (St.3-5); Area 3, Hamanaka Bay facing Kiritappu Wetland (St. 6-8); Area 4, Hamanaka Bay, apart from Kiritappu Wetland and directly affected by Coastal Oyashio Water (St.9-11). Biwase Bay and Hamanaka Bay are geographically divided by peninsula of Cape Kiritappu. Stations were along the 5-m isobath except for St.1 (1.8 m). Before seawater sampling, seawater temperature and salinity were measured with a conductivity, temperature, and depth (CTD) sensor (RINKO profiler ASTD102; JFE Advantech, Nishinomiya, Japan). Next, samples for the measurement of nutrient concentration, total and size-fractionated Chl *a* concentrations, cell density and composition of diatoms, absorption coefficient of colored dissolved organic matter (CDOM) were collected from the surface using an acid-cleaned bucket.

### Nutrient concentration

Samples for nutrient analysis (50 mL) were filtered through a 0.45-μm syringe filter and stored at −80 °C in an ultra low temperature freezer prior to analysis. Nitrite (NO_2_-N), nitrate (NO_3_-N), ammonium (NH_4_-N), phosphate (PO_4_-P) and silica (SiO_2_-Si) concentrations were measured with a BL-Tec autoanalyzer (QuAAtro). Nutrient concentrations were quality controlled against reference material for nutrients in seawater (RMNS) (KANSO TECHNOS CO., Ltd., Osaka, Japan) (Aoyama et al., 2012).

### Absorption coefficients of colored dissolved organic matter

Seawater was filtered into acid-cleaned amber glass bottles to measure the absorption of colored dissolved organic matter (CDOM). Filtration required using a hand-operated vacuum pump (<0.013 MPa) to push water through a 47-mm Nuclepore membrane (0.2-μm pore size). CDOM optical density (OD_CDOM_(λ)) was measured in a 10-cm quartz cylinder cell from 350 to 750 nm in 1-nm increments using a double beam spectrophotometer (UV-2600; Shimadzu, Kyoto, Japan). Milli-Q water was used as a reference. We minimized the effects of temperature and salinity on the absorbance reading of the samples by averaging the OD_CDOM_(λ) value over a 5-nm interval. Measurements around 685 nm were assumed to be 0, and the OD_CDOM_(λ) spectrum was subsequently shifted to account for the effects (Babin et al., 2003). The following equation converted our measured absorbance values into absorption coefficients of CDOM, *a*_CDOM_(λ):

(1) $$a_\rm {CDOM}(\lambda) = 2.303{\cdot}OD_{CDOM}(\lambda)/0.1$$

where 2.303 is a factor for converting between log_10_ and the natural logarithm, and 0.1 is the optical pathlength (m).

### Chlorophyll *a* concentration

The seawater samples (126 mL) for total Chl *a* concentration were filtered onto 25-mm Whatman GF/F filters (~0.7-μm pore size) under vacuum pump (<0.013 MPa). The seawater samples (200–250 mL) for size-fractionated Chl *a* concentrations were also filtered onto 47-mm Nuclepore membranes (10- or 2-μm pore size) or Whatman GF/F filters (~0.7-μm pore size) with a hand-operated pump (<0.013 MPa). The filters were soaked in 6 mL of *N,N*-dimethylformamide (DMF) in a glass cuvette at −20 °C for more than 24 h (Suzuki & Ishimaru, 1990). Chl *a* concentration was determined using a Turner Designs 10-AU fluorometer with the non-acidification method of Welschmeyer (1994). In this study, the fractions of three size classes were defined as micro-sized (>10 µm), nano-sized (2–10 µm), and pico-sized (approximately 0.7–2 µm) Chl *a* concentration to the total Chl *a* levels. Size-fractionated Chl *a* concentrations were analyzed only for Areas 2, 3 and 4.

### Identification and enumeration of diatoms

The seawater (250 mL per station) collected from each station was fixed in 10% formalin immediately after sampling and stored in a cool, dark location until use. For microscopic observation, particles were settled to the bottom of 10 mL volume of Utermöhl chamber following Utermöhl (1958). Diatoms were identified at the genus level and the number of cells were counted under an inverted microscope (CKX53; OLYMPUS, Shinjuku City, Tokyo, Japan) with 400× magnification. Stations 2, 4, 7, and 10 were selected as representative stations for each of 4 areas, because those stations were in the middle of areas and along the isobath. Diatoms of those four stations were identified and counted for three replicated subsamples of 10 mL.

### Statistical analysis

Statistical analyses were conducted for each environmental variable (water temperature, salinity, CDOM, Chl *a*, nutrients) from all stations of each area (Area 1: St. 1 and 2, Area 2: St. 3, 4, and 5, Area 3: St. 6, 7, and 8, Area 4: St. 9, 10, and 11), and for the number of counted diatom cells of representative stations (St. 2, 4, 7, and 10) using one-way analysis of variance (ANOVA) followed by Tukey’s HSD. Cluster analysis based on the cell density of diatoms at the genus level was carried out using Unweighted Pair Group Method with Arithmetic mean (UPGMA) based on a Bray-Curtis matrix. Statistical analyses were conducted with the R software v.4.0.5 (R Core Team, 2018).

## Results

### Environment of sampling sites

**Figure 2:**
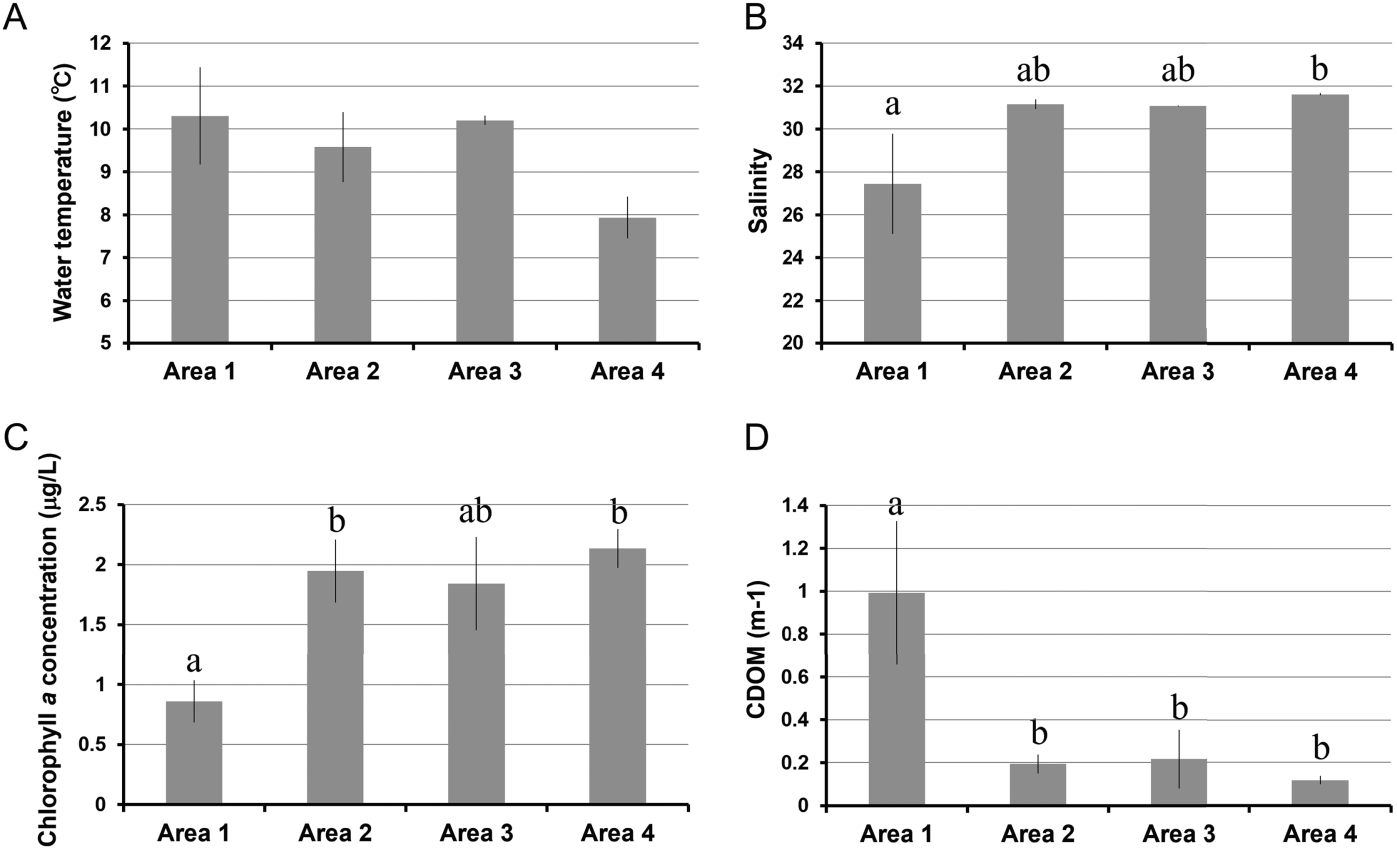
Environmental variables measured in four areas of Biwase Bay and Hamanaka Bay. (A) Water temperature, (B) salinity, (C) chlorophyll *a*, and (D) colored dissolved organic matter. Data are expressed as the mean ± standard deviation (*n* = 3) except for Area 1 (*n* = 2). Different letters indicate significant differences (Tukey HSD).

**Figure 3:**
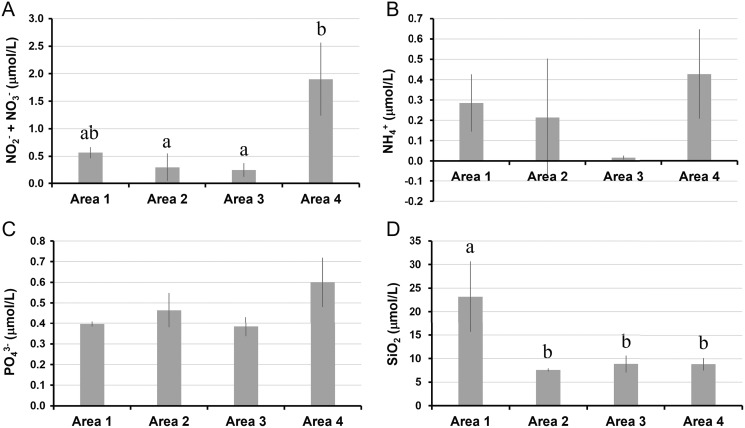
Nutrient concentrations measured in four areas of Biwase Bay and Hamanaka Bay. (A) Total amount of nitrite and nitrate (NO_2_^−^+ NO_3_^−^), (B) ammonium (NH_4_^+^), (C) phosphate (PO_4_^3−^), and (D) silica (SiO_2_). Data were expressed as the mean ± standard deviation (*n* = 2–3). Different letters indicate significant differences (Tukey HSD).

The water temperature in Area 4 was relatively lower than those in other areas. Even though there was a significant spatial variation by ANOVA, the post-hoc test showed no significant difference between each pair of areas (Table 1, Fig. 2A). Salinity was significantly lower in Area 1 than Area 4, while no significant difference was found in other pairs of areas (Fig. 2B). Chlorophyll *a* concentration ranged from 0.68 µg/L to 2.33 µg/L. The lowest Chl *a* concentration was found in Area 1 (Fig. 2C). The *a*_CDOM_(443) was significantly higher in Area 1 than in the other three areas (Fig. 2D). The total amount of nitrite and nitrate concentrations was significantly higher in Area 4 than in Areas 2 and 3 (Fig. 3A). In contrast, neither ammonium nor phosphate concentrations showed significant differences among areas (Figs. 3B, 3C). Silica concentration in Area 1 was significantly higher than in the other areas (Fig. 3D).

**Table 1:** Results of one-way ANOVA testing among-area variation in environmental and biotic variables.

Variables	F	*df*	*p*
Temperature	4.40	3	**0.049**
Salinity	5.18	3	**0.034**
Chlorophyll *a*	6.34	3	**0.021**
*a*_CDOM_ (443)	9.03	3	**0.008**
Nitrite and nitrate	7.93	3	**0.012**
Ammonium	1.38	3	0.325
Phosphate	2.75	3	0.122
Silica	6.50	3	**0.020**
Diatom cells	173.90	3	**<0.001**

**Note:**

Bold letters indicate the significant differences of variables among areas (*p* < 0.05).

### Size-fractionated chlorophyll a concentrations

**Figure 4:**
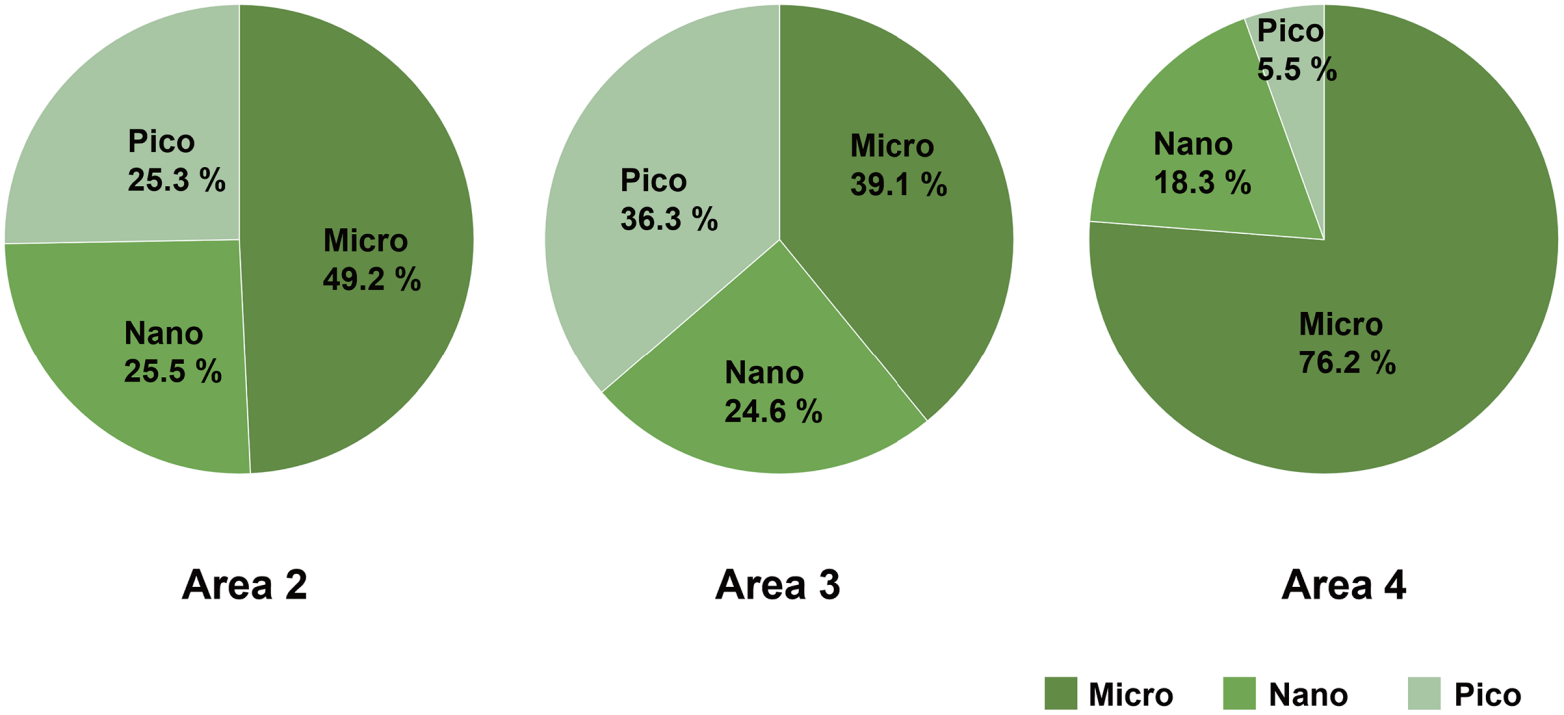
The composition of size-fractionated Chl *a* concentration (microplankton, nanoplankton and picoplankton) at three areas. Data not available for Area 1 due to absence of water sample.

Micro-sized (>10 µm) phytoplankton dominated the total phytoplankton community in Area 2, while the composition of nano- (2–10 µm) and pico-sized (0.7–2 µm) phytoplankton accounted for 25.5% and 25.3%, respectively. In Area 3, the composition of micro- (39.1%), nano- (24.6%), and pico- (36.3%) sized phytoplankton were similar. On the other hand, the predominance of micro-sized phytoplankton (76.2%) was found in Area 4 (Fig. 4).

### Composition of diatom

**Figure 5:**
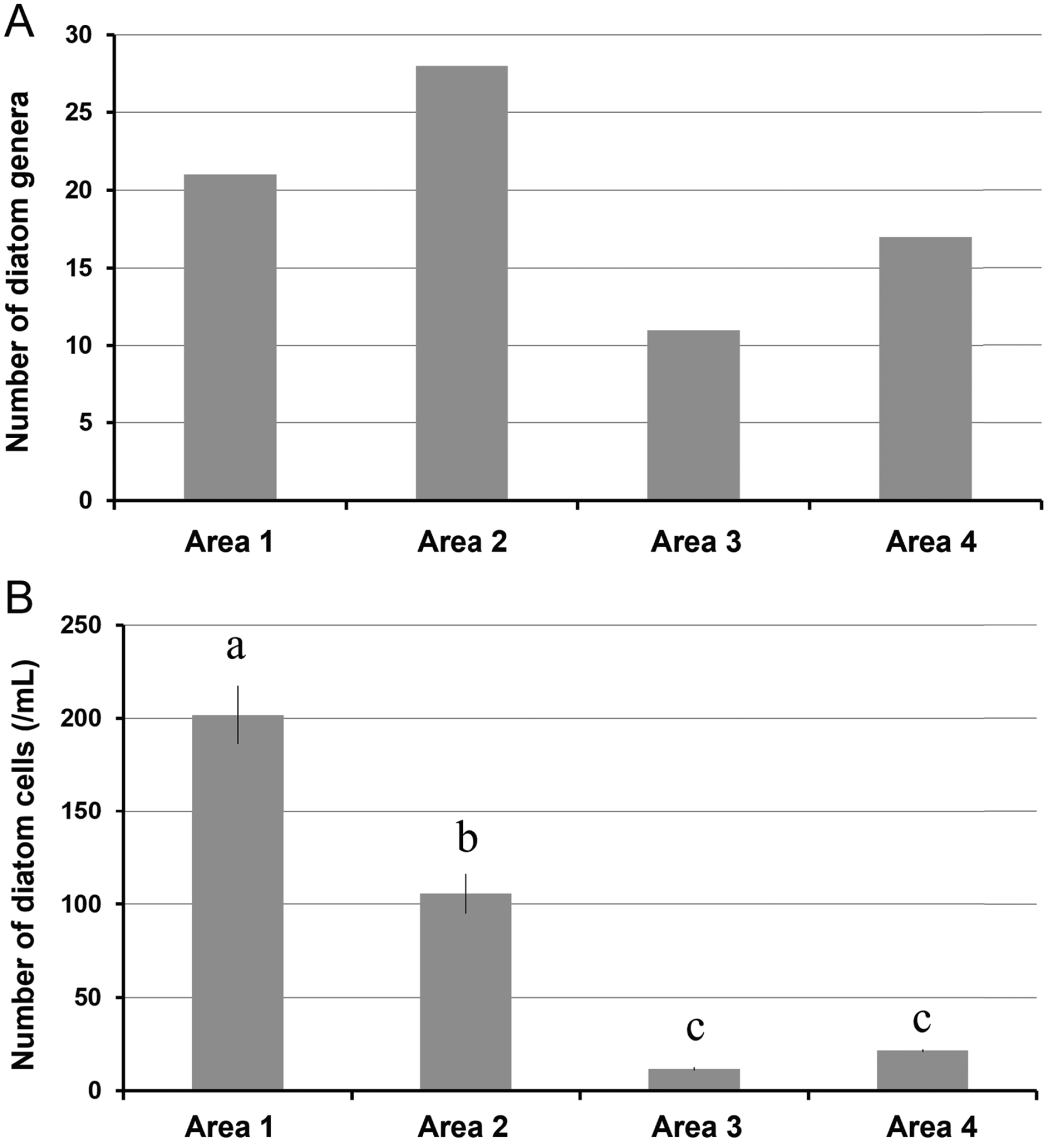
The number of diatom genera observed (A), and the number of diatom cells counted (per 1 mL volume) (B) in each station (St.2, 4, 7, and 10) representing area. Data were expressed as (A) total number of genera from triplicate observation and (B) the mean ± standard deviation. Different letters indicate significant differences (Tukey HSD).

**Figure 6:**
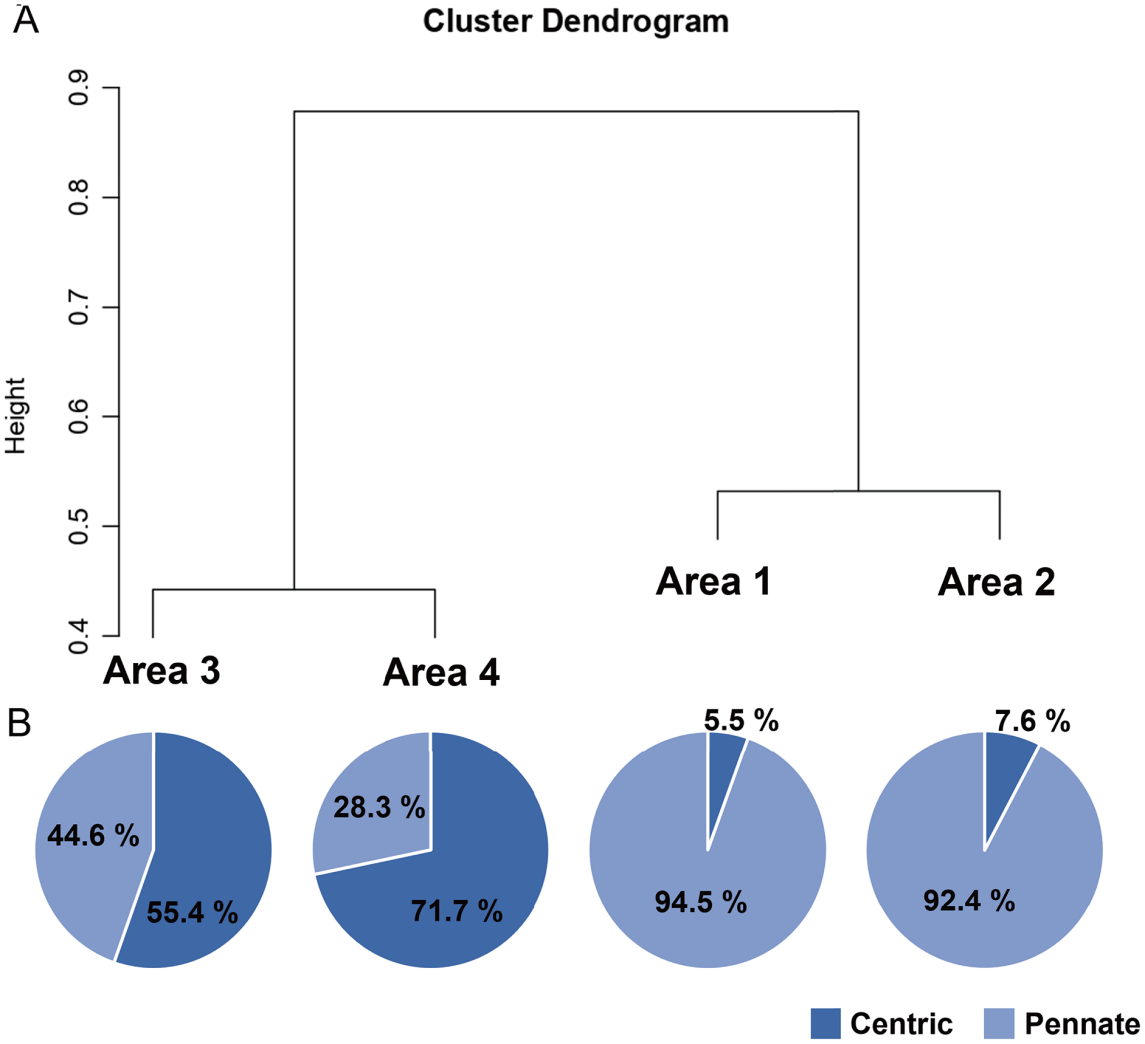
UPGMA dendrogram based on the Bray-Curtis matrix (A), and the relative abundance of centric and pennate diatoms in each area (B).

A total of 34 genera were observed from four different areas. Diatom composition was more diverse in Biwase Bay than in Hamanaka Bay. There were 21 and 28 diatom genera counted in Areas 1 and 2, respectively, whereas 11 and 17 genera were found in Areas 3 and 4 (Table 2, Fig. 5A). The abundance in Area 1 was significantly higher than those in the other three areas, and diatoms in Area 2 were significantly more abundant than Areas 3 and 4. There was no significant difference between Areas 3 and 4 (Fig. 5B). Cluster analysis based on the diatom genera and their cell densities classified the areas into Biwase Bay (Areas 1 and 2) and Hamanaka Bay (Areas 3 and 4) (Fig. 6A). In addition, pennate diatoms were more abundant in Biwase Bay, while centric diatoms were abundant in Hamanaka Bay (Fig. 6B). The most dominant diatom genus was *Cocconeis* in Biwase Bay (88.7% at Area 1, 56.9% at Area 2), and *Thalassiosira* in Hamanaka Bay (42.8% at Area 3, 57.9% at Area 4) (Table 2).

**Table 2:** List of diatom genera observed by light microscope and cell numbers (cells/L) in four stations (St.2, 4, 7, and 10) representing each area of Biwase Bay and Hamanaka Bay.

		Biwase Bay		Hamanaka Bay	
Name of diatom genera		Area 1	Area 2	Area 3	Area 4
		(St. 2)	(St. 4)	(St. 7)	(St. 10)
Centric	*Actinocyclus*		67		67
	*Actinoptychus*	667	67	333	167
	*Arachnoidiscus*		33		
	*Biddulphia*		67		
	*Ehrenbergia*	1,700	933	100	467
	*Hyalodiscus*	867			167
	*Melosira*	33			
	*Odontella*	367			
	*Paralia*	2,467	1,167	500	2,233
	*Thalassiosira*	1,533	2,667	3,367	8,467
	*Triceratium*		33		
Pennate	*Achnanthes*	33			
	*Amphora*	267	767		100
	*Cocconeis*	123,667	37,267	200	733
	*Cosmioneis*		67		
	*Cylindrotheca*		133		
	*Delphineis*	300	2,767	1,700	1,800
	*Diatoma*		133		
	*Diploneis*	100	1,500	67	67
	*Entomoneis*		233		
	*Fallacia*	500	4,300		33
	*Fragilaria*		33		
	*Gyrosigma*	67	67	33	100
	*Hyalosynedra*	67			
	*Licmophora*		167		
	*Navicula*	4,600	9,233	1,267	1,067
	*Nitzschia*	1,667	633	100	100
	*Planothidium*	567	1,333		
	*Pleurosigma*	67	33		500
	*Rhaphoneis*	33	1,767	100	33
	*Surirella*	33			
	*Tabularia*		233		
	*Trachyneis*		33		
	*Tryblionella*		33		33
Subtotal of diatom cells (/L)		139,600	65,767	7,767	16,133
Number of genera occurring		21	28	11	17

## Discussion

We predicted that the ocean environment of study area would differ geographically between Biwase Bay (Areas 1, 2) and Hamanaka Bay (Areas 3, 4). However, the actual environment was more complicated: the water temperature of Area 4 was relatively lower than the other areas (Fig. 2A), reflecting the influence of the cold Coastal Oyashio Water (COW). The influence of COW, characterized as lower than 3 °C and 33.0–33.3 (Ohtani, 1971), has been reported widely from eastern Hokkaido (Kasai et al., 1997) to northeastern parts of Honshu, Japan (Ishizu et al., 2017). On the contrary, Area 1 with the lowest salinity is strongly affected by the river discharge from Kiritappu Wetland as it is located near the Biwase River mouth (Fig. 2B). The *a*_CDOM_(443) and silica concentration in Area 1 were significantly higher than other areas, suggesting that the river discharge mainly affects only the southern part of Biwase Bay (Figs. 2D, 3D). Ba et al. (2018) conducted stable isotope analysis of nitrogen and carbon for oceanic particle organic materials which demonstrated how the ratios of oceanic organic matter were different from those of riverine organic matter. Our results are in agreement with their findings.

The Chl *a* concentration was the lowest at Area 1 (Fig. 2C), which is the nearest site to the river mouth. The low concentration does not support previous conclusions of a positive relation between increased river discharge and Chl *a* concentration in the adjacent coastal area (Lihan et al., 2011; Morey, Dukhovskoy & Bourassa, 2009; Wysocki et al., 2006). Typically, Chl *a* concentration increases due to the nutrient input from the river discharge (Masotti et al., 2018). However, the Biwase River system is unique because water must first pass through the pristine Kiritappu Wetland, a buffer against terrestrial matter (Fig.1), and also because human impact is minor in this town due to the low population size (6,249 in 2014; from the homepage of Hamanaka Town, https://www.townhamanaka.jp/). Therefore, nitrate and phosphate concentrations were not significantly higher at Area 1 (Figs. 3A–3C). The discrepancy between previous and present studies might be due to the difference of surrounding terrestrial environment. Besides of the low nutrient levels, the low Chl *a* concentration of Area 1 may be the result of the narrow mouth (<100 m) of the Biwase River creating a high velocity current. Quantity of plankton is negatively related to the current speed (Brook & Rzòska, 1954). In addition, the relatively lower concentration of Chl *a* at Area 1 might be caused by a lesser influence of COW with high nutrients (Kasai et al., 1997). The results of size-fractionated Chl *a* concentration suggests that Area 4 is more oceanic than Areas 2 and 3 because micro-sized phytoplankton were predominant (Fig. 4). The relatively high value of total phosphate, nitrite, and nitrate in Area 4 might affect the dominance of micro-sized phytoplankton (Figs. 3A, 3C).

The effects of geographical division appeared to have a greater impact on diatom flora than water quality related to COW and river discharge from the wetland. Diatom composition in Biwase Bay differed from those in Hamanaka Bay. Taxa richness and abundance of diatom were higher in Biwase Bay than in Hamanaka Bay (Fig. 5). In addition, the cluster analysis classified the four areas into two distinct groups: Biwase Bay and Hamanaka Bay. Pennate diatoms were predominant in Biwase Bay while centric diatoms were predominant in Hamanaka Bay (Fig. 6). The variation in diatom composition and distribution by area has been reported in several research. Van de Vijver et al. (2020) showed that diatom abundance could be separated into distinct groups that are strongly aligned with different countries (Croatia, America, Greece, South Africa). Wachnicka et al. (2010) compared diatom distributions in freshwater, mangrove, and estuaries of Florida Bay in dry and wet season, and found that the differences in communities were significant among sites for both seasons. In those two studies, sampling sites were scattered at broad spatial scale such as across different continents and distinguishable ecosystems. At a finer scale, Trábert et al. (2020) studied benthic diatoms in the Danube River, finding that the dissimilarity index based on species abundance aligned with the different topographies based on the separation between upstream and downstream. Our findings also suggest that diatom composition can vary among water divided by land at the scale of 10–15 km.

Of the 34 genera of diatoms (11 centric and 23 pennate) present in the present study, 11 genera were found in all four areas (Table 2). Seventeen genera were only observed in Biwase Bay (Areas 1, 2), while no genus was completely unique to Hamanaka Bay (Areas 3, 4). The high species richness of Biwase Bay was possibly associated with benthic and epiphyte diatom flora from Kiritappu Wetland exported by Biwase River. Though the diversity was lower in Hamanaka Bay (Fig. 5), Chl *a* concentration was not (Fig. 2C) because larger-sized oceanic genera such as *Thalassiosira* were predominant in Hamanaka Bay (Table 2). In comparison with other studies on diatom flora, our observations of 17 genera also occurred in other wetlands of eastern Hokkaido. Sawai (2001) reported that pennate genera (13 out of 23 (56.5%)) showed higher accordance than centric genera (4 out of 11 (36.4%)). In contrast, centric genera (9 out of 11 (81.8%)) showed higher accordance than pennate genera (15 out of 23 (65.2%)) from a study by Kasim & Mukai (2006) who had 24 genera overlapping our work. This study was centered on the Akkeshi-ko estuary, which is located adjacent to Biwase and Hamanaka Bays and is also affected by COW.

## Conclusions

Diatom composition along the nearshore areas of Biwase Bay and Hamanaka Bay, eastern Hokkaido, Japan, showed significant spatial variation in abundance and taxa within a spatial extent of 10–15 km. Environmental variables such as temperature, salinity and nutrient contents, and geographical feature of the coast were related to our observed variation. The variation in the environmental factors appear to be strongly affected by both the Coastal Oyashio Water and terrestrial input by Biwase River and surrounding Kiritappu Wetland. Geographical influences are related to the historical connectivity of pelagic waters that are now divided by the peninsula laying between Biwase and Hamanaka Bays. Although this study is based on just a snapshot of data, it is the first dataset collected for Hamanaka coastal water. The obtained data can be used as baseline data for future research, including the evaluation of impacts of ongoing climate change on coastal ecosystems, which is expected to heavily affect the coastal ecosystems and biodiversity of this region (Sudo et al., 2020).

## Supplemental Information

10.7717/peerj.13705/supp-1 Supplemental Information 1 Raw data of water quality and tables.

## Additional Information and Declarations

### Data Availability

The following information was supplied regarding data availability: The raw measurement data of environmental and biotic variables, nutrient concentrations are available in the Supplemental File.
